# The Effects of Scheduled Smoking Reduction and Precessation Nicotine Replacement Therapy on Smoking Cessation: Randomized Controlled Trial With Compliance

**DOI:** 10.2196/39487

**Published:** 2023-06-20

**Authors:** Paul M Cinciripini, Jennifer A Minnix, Jason D Robinson, George Kypriotakis, Yong Cui, Janice A Blalock, Cho Y Lam, David W Wetter

**Affiliations:** 1 Department of Behavioral Science University of Texas MD Anderson Cancer Center Houston, TX United States; 2 Department of Population Health Sciences University of Utah Salt Lake City, UT United States

**Keywords:** gradual reduction, scheduled smoking, nicotine replacement therapy, smoking cessation, compliance, mobile phone

## Abstract

**Background:**

Smoking remains a major public health problem, and it is important to provide a variety of efficacious and appealing options to encourage smokers to quit smoking. Scheduled smoking is a method of gradual reduction, preparing smokers to quit by systematically reducing cigarette consumption according to a predetermined schedule that increases the time between cigarette consumption. Gradual reduction may be preferred to abrupt quitting, but the efficacy of this cessation approach is unclear.

**Objective:**

This study aims, first, to evaluate the overall effectiveness of scheduled smoking alone, or in combination with precessation nicotine replacement therapy (NRT), versus standard NRT starting on the quit date with no prior smoking reduction and, second, to evaluate the impact of schedule compliance on the effectiveness of the intervention.

**Methods:**

A total of 916 participants recruited from the Houston metropolitan area were randomly assigned to 1 of the following 3 groups: scheduled smoking plus a precessation nicotine patch (n=306, 33.4%), scheduled smoking only with no precessation patch (n=309, 33.7%), and enhanced usual care (n=301, 32.9%) control. The primary abstinence outcomes were carbon monoxide–verified, self-reported, 7-day point prevalence abstinence at 2 and 4 weeks after the quit date. Unadjusted and adjusted logistic regression analyses were performed to evaluate the intervention effect. Scheduled smoking was implemented using a handheld device for 3 weeks before quitting. This trial was not registered because data collection began before July 1, 2005.

**Results:**

Results for the first aim showed no overall differences in abstinence among the 3 groups in both the unadjusted and adjusted models. However, the results for the second aim showed a clear effect on abstinence by schedule compliance at 2 and 4 weeks and 6 months after quitting (odds ratio [OR] 2.01, 95% CI 1.31-3.07), 4 weeks (OR 1.58, 95% CI 1.05-2.38), and 6 months (OR 1.68, 95% CI 1.04-2.64), with the differences at 2 and 4 weeks after quitting being the most robust. We also found that scheduled smoking was related to a reduction in nicotine withdrawal, negative affect, and craving when compared with the controls.

**Conclusions:**

Scheduled smoking, when combined with precessation use of NRT, can result in significantly higher abstinence rates than usual care (abrupt quitting with NRT), particularly in the early postquit phase (2 and 4 weeks after cessation) when smokers are compliant with the procedure. Scheduled smoking also produced a better overall quitting experience by reducing symptoms of nicotine withdrawal and craving, in comparison with usual care, which could encourage future quit attempts. Studies in this area should focus on the use of counseling or other methods to improve adherence.

## Introduction

Smoking remains a major public health problem, and it is currently responsible for approximately 6 million premature deaths every year worldwide and is predicted to cause approximately 8 million deaths annually by the year 2030 [1]. Tobacco plays a causal role in at least 15 types of cancer [2,3], accounts for approximately 30% of the attributable risk for overall cancer mortality, and is responsible for >87% of all lung cancer–related deaths, 61% of chronic obstructive lung disease–related deaths, and 32% of heart disease–related deaths. In the United States, cigarette smoking accounts for as much as US $333 billion annually, including up to US $176 billion in direct medical care costs and US $151 billion in lost productivity from premature deaths [4].

Approximately 70% of smokers report that they want to quit smoking every year [5], and although 55% of the smokers report having made a serious attempt, annually, only approximately 8% achieved some level of success [6]. In fact, the annual success rate of any one individual attempt at quitting on one’s own (without treatment) is ≤5% [7]. Subsequent quitting attempts after an initial attempt are extremely common, and for many smokers, multiple attempts are essential to achieve long-term success [8]. The overall impact of successful smoking cessation on population health is substantial, including reducing the prevalence, morbidity, or mortality of 15 tobacco-related cancers, stroke, cardiovascular disease, diabetes, chronic obstructive pulmonary disease, and rheumatoid arthritis, as well as reducing the cost of associated health care expenditures [4,9]. It is important to provide a variety of efficacious and appealing quitting options to encourage smokers, especially those who have tried to quit repeatedly without success, to make another quit attempt.

An assortment of pharmacological and behavioral treatment strategies has been shown to increase cessation rates in adult smokers [10-12]. Traditionally, formal guidelines primarily recommend that people stop smoking abruptly on a future (prespecified) quit day but smoke normally up until this day [10,13]. An alternative method is to gradually reduce the amount of tobacco smoked (usually over the course of a few weeks) before quitting completely on the quit day. Gradual reduction methods have been implemented in several ways, such as limiting the time (or situations) in the day when smoking occurs (*smoke-free periods*) [14], using nicotine replacement therapy (NRT) or other types of pharmacotherapy to replace or help cope with cigarettes not smoked [15,16], setting goals to reduce by a certain number or percentage of cigarettes per day [17], or reducing smoking before quitting without a specific plan [18].

Scheduled smoking is a gradual reduction method of preparing to quit smoking in which smokers reduce their smoking level by systematically increasing the time between cigarette consumption using a predetermined schedule [19-22]. This is accomplished by spreading the increasingly diminishing number of cigarettes evenly over the waking hours so that as fewer cigarettes are smoked as the time between each cigarette gradually increases. This strategy may help to begin the process of decoupling smoking behavior with specific smoking triggers because the smoking schedule dictates when the cigarette is smoked and prohibits off-schedule smoking. As a result, smokers may not be able to smoke when they want to and might be asked to smoke at times that are not ideal or convenient. Scheduled reduction may compel smokers to use coping strategies to overcome the urge to smoke even before the quit day [23], skills that may ultimately contribute to long-term abstinence.

NRT was formulated to partially replace the nicotine that would have been consumed in a cigarette without the additional harmful components of combustible tobacco [24], thereby reducing the severity of withdrawal symptoms and increasing the likelihood of sustained abstinence [25]. The effectiveness of NRT has been well established [26,27], and its use generally doubles the likelihood of successful cessation. Despite this, overall abstinence rates among smokers who use NRT, although consistent, are relatively modest (20%-30%) [28,29]. NRT was originally designed to be used after quitting smoking (starting on the quit day), but studies have investigated its use during the precessation period, when a smoker often attempts to gradually reduce before the quit day.

The public health impact of smoking cessation treatments (ie, the number of smokers who successfully quit) depends on the uptake of treatments in the smoking population as well as their effectiveness in real-world use [30]. Previous smoking cessation research has demonstrated that adherence to treatment components in both the delivery from the provider’s perspective (fidelity) and the adherence to dosing, schedules, goals, etc from the client’s perspective can affect success in increasing abstinence [19,23,31-33]. Evidence from a meta-analysis of clinical studies suggests that the lack of adherence to NRT regimens undermines their effectiveness [34]. Relatively higher smoking cessation rates have been reported in clinical trial participants (50%-60%) [27] compared with participants of population-based studies (20%-30%) [28,29]. Not surprisingly, the rate of adherence to NRT was found to be >2-fold higher in participants of clinical trials than in participants of population-based studies [33]. Similarly, as noted earlier, differences in scheduled smoking adherence may have contributed to differences in the effect of scheduled smoking on abstinence in previous studies [19,20]. Hence, the examination of intervention effectiveness, particularly when ≥2 interventions are combined (eg, precessation of NRT and scheduled smoking), should take compliance into account.

This study aimed to (1) evaluate the overall effectiveness of scheduled smoking alone, or in combination with precessation NRT, versus standard NRT starting on the quit date with no prior smoking reduction and (2) assess and evaluate the impact of schedule compliance on the effectiveness of the intervention.

## Methods

### Participants

Participants were recruited from the Houston metropolitan area using newspaper and billboard advertisements. A total of 1773 smokers were screened via telephone for study eligibility. Only healthy smokers who were interested in quitting smoking, had no uncontrolled medical illness, were fluent in English, and smoked at least 10 cigarettes per day were included in the study. Individuals were excluded if they were taking psychotropic medications, met the criteria for a current psychiatric disorder, or were involved in current smoking cessation activities. Of the 1773 smokers, 1035 (58.38%) met all the criteria and were invited to enroll in the study. However, of the 1035 smokers, 119 (11.5%) did not attend the baseline visit, resulting in 916 (88.5%) participants being randomized and issued a study device. Of the 916 randomized participants, 96 (10.5%) returned the device unused and thus were not exposed to treatment and were therefore excluded from the outcome analyses.

Participants could earn up to US $115 in gift certificates by completing study procedures. All participants received a US $50 gift certificate for the completion of visits through the end-of-treatment visit and a US $15 gift certificate for completion of the 2 follow-up visits. Participants also received a total of US $5 to US $50 in gift certificates based on completion of random mood and smoking assessments generated by their computer while in the study.

### Overall Design

The protocol was approved by the institutional review board of The University of Texas MD Anderson Cancer Center. This trial was not registered because the data collection began before July 1, 2005. Data collection for this study was conducted from 2000 to 2006, before the advent of smartphones. The data analysis for this study began in 2018. The data from this study and the customized program provided the basis for a new scheduled smoking smartphone app designed to run on the current Android platform. This program is currently being tested and will be the subject of a future study. A clinical version (nonresearch) will be made available for download following testing.

Participants were randomly assigned to 1 of the following 3 groups: scheduled smoking plus a precessation nicotine patch (SSNP), scheduled smoking only with no precessation patch (SS), and enhanced usual care (EUC) control. The groups were stratified by sex, race, cigarettes smoked per day, and depression history. The study was divided into 4 phases: baseline (2 days), precessation date intervention (21 days; SSNP and SS groups only), postquit date intervention phase 1 (14 days; including the quit date), and postquit date intervention phase 2 (8 weeks). During the baseline period, all groups were instructed to smoke ad libitum and use a handheld device (HD) to record their smoking, and to receive other programming, as described in the section below. During the precessation period, both scheduled smoking groups (SSNP or SS) were exposed to a 21-day computerized intervention delivered on the HD, designed to progressively reduce daily cigarette consumption before their quit date. The SSNP group also began using a nicotine patch simultaneously with the reduction schedule. The EUC group did not receive a precessation patch or cigarette reduction intervention. During the postquit date intervention (phase 1), which included the quit date, all groups used the nicotine patch and HD to continue recording smoking behavior and receive other programming, as noted in the Scheduled Smoking section below. During the postquit date intervention (phase 2), all groups stopped using the HD but continued using the nicotine patch for an additional 8 weeks.

### Scheduled Smoking

During the 3-week precessation period, smokers in both the SSNP and SS groups were instructed to smoke at scheduled times only. The computer-assisted scheduled smoking intervention application, active on the HD for the SSNP and SS groups only, prompted the smoker to smoke only at specific times during the day. Over a period of 3 weeks, the program gradually increased the interval between prompted cigarettes (ie, the intercigarette interval) such that only 3 to 4 cigarettes were scheduled to be consumed the day before the quit date. The reduction schedule was implemented using the following algorithm: (1) using the daily smoking mean and wake time derived from the participants’ self-report at baseline, the program calculated an intercigarette interval by dividing the average number of minutes awake per day by the reduced percentage of the smoker’s average daily smoking frequency. (2) For days 1 to 12, the baseline smoking rate was reduced by 15% every 3 days, and for days 13 to 21, a 10% reduction every 3 days was used (this process typically resulted in a 75% reduction in the baseline smoking rate by the beginning of the third week of the schedule and corresponding progressively longer intercigarette intervals). (3) The process outlined in step 2 was used to determine the maximum allowable cigarettes for that period; however, a 15-minute delay to the first cigarette of the day was added to the schedule each week, and because of this delay, any cigarette consumption that would have occurred past the participants’ bedtime were not added to the schedule. Moreover, once the smoking frequency was reduced to 3 to 4 cigarettes per day, no further reductions took place until the quit date, when zero cigarettes were expected. (4) All smoking times were adjusted to fall on the nearest quarter within the hour. A sample schedule over the 3-week scheduled smoking period is provided in Figure S1 in Multimedia Appendix 1 [1-22,29-58].

### Visit Structure

Participants in the SSNP and SS groups attended 9 in-person visits as follows: baseline, 3 weeks precessation, 2 weeks precessation, 1 week precessation, 2 days after quitting, 2 weeks after quitting, 4 weeks after quitting, 6 months after quitting, and 12 months after quitting.

Participants in the EUC group had no precessation scheduled smoking or reduction intervention and hence attended only 6 in-person visits (baseline, 2 days after quitting, 2 weeks after quitting, 4 weeks after quitting, 6 months after quitting, and 12 months after quitting).

At the baseline visit (approximately 45 minutes), participants were instructed on the use of the HD, which included watching a short video explaining the group-specific and general features of the program, and were assessed on their understanding of device use. All other visits (approximately 20 minutes) were used to obtain questionnaire information, assess abstinence, and download data from the HD. No smoking cessation counseling was provided during these visits.

### NRT Overview

During the 21-day precessation date intervention period (Figure S2 in Multimedia Appendix 1), smokers in the SSNP group began wearing a 14-mg nicotine patch on the first day of scheduled smoking, which was continued during the 3-week precessation period. Smokers in the SS group did not receive nicotine patches during the precessation period. During the postquit date period (phase 1), smokers in both the SSNP and SS groups began using the 21-mg patch on their quit date regardless of whether they quit early or not. Smokers in the EUC group began using the active 21-mg patch on the quit day, which occurred 2 days after their baseline visit.

During the 10 weeks following the target quit date, all the participants continued to use the nicotine patch. The participants used the 21-mg patch for 6 weeks, followed by the 14-mg patch for 2 weeks, and finally a 7-mg patch for 2 weeks.

### HD: Study Device and Programming

The study device was the Cassiopeia Pocket PC E-125 (Casio Computer Co Ltd), an HD that was a precursor to today’s smartphones (Figure S3 in Multimedia Appendix 1). The device was used to deliver the custom programmed scheduled smoking intervention in the 2 scheduled smoking groups, along with assessments and routines common to all groups. To provide a control for HD use and support internal validity, those in the EUC group were issued the same HD with all the functionality provided to the scheduled smoking groups but without the scheduled smoking routine. The provision of the HD to the control group distinguished this group from a “usual care control” because the programming provided some “enhanced” features unrelated to scheduled smoking, which were designed to provide cessation tips, conduct smoking assessments, foster relapse prevention, and renew quit attempts following a relapse (refer to HD functionality description and Figure S3 both in Multimedia Appendix 1).

Other applications on the device were disabled. All groups received the study device on the day of randomization to treatment. The 2 scheduled groups used the device for a total of 37 days, including 2 days before the quit date (precessation), 3 weeks precessation, and 2 weeks after cessation (from the initial quit date). The EUC group used the device for 2 days precessation and 2 weeks after cessation.

The HD shared common functionality across groups, including an embedded electronic cessation tip guide; programmed assessments of smoking behavior, mood, and other conditions delivered at both fixed (daily diary) and random times (ecological momentary assessments); and user-initiated recording of actual smoking, temptations (urges) to smoke, and quit attempts. The wake-up time and bedtime were recorded/confirmed daily (Figure S2 in Multimedia Appendix 1). The tip guide consisted of a series of hyperlinks embedded in the program. Each smoker also received a printed version of the tip guide at randomization (Figure S4 in Multimedia Appendix 1).

The schedule smoking was initiated when the HD signaled a smoking interval by flashing a display and sounding an alarm. A sample screenshot for the first day of scheduled smoking is shown in Figure S5 in Multimedia Appendix 1. The program included routines (Figure S6 in Multimedia Appendix 1) to allow for early quitting in the scheduled groups (ie, making a quit attempt before the quit date). During the 2 weeks immediately following the quit date, participants in all 3 groups were provided with a relapse management routine (Figure S7 in Multimedia Appendix 1) if they indicated that they had smoked (even a puff) on the previous day during the wake-up routine or entered that they smoked a cigarette at any time (Multimedia Appendix 1 provides additional details on methods and device).

### Measures

#### Primary Care Evaluation of Mental Disorders

At baseline, the participants were administered the Primary Care Evaluation of Mental Disorders (PRIME‑MD) [59]. The PRIME‑MD screened for the 5 major mental health disorders (Diagnostic and Statistical Manual of Mental Disorders, fourth edition) most commonly encountered in the general population (mood, anxiety, somatoform, alcohol, and eating disorders) using a 25-item patient self-report questionnaire and a structured interview. The interview was used to follow-up on positive responses on the patient questionnaire and determine if the Diagnostic and Statistical Manual of Mental Disorders, fourth edition criteria were met for these current psychiatric disorders. Administration of PRIME-MD lasted approximately 10-15 minutes.

#### Demographic, Health, and Smoking History Questionnaires

Participants completed a questionnaire to assess the demographics; medical and health history of respondents and their families; current medications; alcohol intake; and smoking history, including years smoked, previous quit attempts, relapse, current smoking rate, and other nicotine or tobacco use. This questionnaire has been used in a number of our studies on smoking cessation and relapse [60,61].

#### The Fagerström Test for Cigarette Dependence

The Fagerström Test for Cigarette Dependence (formerly the Fagerström Test for Nicotine Dependence) [62,63] is a 6-item questionnaire that measures nicotine dependence by assessing various components of smoking behavior such as daily intake, difficulty in refraining from smoking, and other aspects related to patterns of intake [63].

The subsequent questionnaires were administered at baseline and at each subsequent in-person visit.

#### The Wisconsin Smoking Withdrawal Scale

The Wisconsin Smoking Withdrawal Scale is a 28-item nicotine withdrawal scale that includes the following 7 subscales: anger, anxiety, concentration, craving, hunger, sadness, and sleep [64].

#### The Positive and Negative Affect Scale

The Positive and Negative Affect Scale comprises two 10-item mood scales: positive affect and negative affect. Various time instructions (eg, today, past few days, past week, and general) have been used with acceptably high α reliability ranging from .86 to .90 for positive affect and .84 to .87 for negative affect [65].

#### The Center for Epidemiologic Studies Depression Scale

The Center for Epidemiologic Studies Depression Scale is a 20-item self-report measure developed to assess depressive symptoms in community (nonclinical) populations [66] and in recent studies on smoking cessation [67]. This scale consists of 4 factors: depressed affect, enervation, lack of positive affect, and interpersonal problems.

#### The Self-Efficacy Scale

The Self-Efficacy Scale is a 14-item scale rating confidence (0-10) at resisting smoking urges within several types of situations [68,69]. The total score has been responsive to both pharmacological and behavioral interventions and has predicted relapse [20,70].

#### Smoking Abstinence

Group differences in the 7-day point prevalence abstinence were assessed at 2 weeks, 4 weeks, 6 months, and 12 months after the quit date. Point prevalence abstinence was defined as a self-report of no smoking during the previous 7 days and an expired carbon monoxide (CO) level of <10 ppm (the standard at the time the study was conducted). Abstinence values were computed in two ways: (1) using an intention-to-treat (ITT) approach in which missing smoking status information at any assessment was imputed as smoking and (2) using a respondent-only (RO) approach in which no imputation for missing information was conducted and abstinence was based on observed data only.

### Data Analytic Plan

#### Compliance With Smoking Reduction

Compliance with the scheduled smoking intervention was assessed in 2 ways. The first method involved a simple calculation of the proportion of scheduled cigarettes smoked on schedule (within 5 minutes of the scheduled time) and the number of cigarettes smoked off schedule. Information for these calculations was obtained directly from the HD. The second method involved assessing the actual level of smoking reduction achieved by examining changes in expired CO between baseline and their last precessation visit (before the to-quit-day). Specifically, a percent reduction score was calculated using the following equation: (expired CO level measured at baseline − expired CO level measured at last precessation visit)/(expired CO level measured at baseline × 100). We prefer the second approach over the one that assesses compliance only via computerized schedule self-report adherence, which may be vulnerable to experimental demand characteristics. That is, participants who desire to present themselves in a better light may make false reports about adhering to the computerized schedule—recording a scheduled cigarette that has not been smoked or failing to record an off-schedule cigarette. In addition, this method accounts for the influence of unscheduled cigarettes on the expired CO.

Using expired CO as a measure of compliance has other advantages over characterizing compliance in terms of the number of cigarettes smoked on schedule. For instance, under the scheduled smoking “rules,” smokers may “miss” a scheduled cigarette as long as they refrain from smoking until the next scheduled cigarette. Therefore, from the point of view of schedule compliance, “missing” a scheduled cigarette is permissible, while smoking an “off-schedule” cigarette to compensate for the loss is not. On the basis of our scheduling algorithm, participants who adhered to their smoking schedule were projected to have reduced their cigarette intake by 75% during the final week of smoking reduction. However, smokers vary in their smoking topography (eg, some inhale deeply while others take shallow puffs), and it is difficult, if not impossible, to determine a reduction in expired CO level that will accurately reflect a 75% reduction in cigarette intake. Therefore, after considering the variation of the time to the last cigarette smoked by participants before they attended their last precessation visit (ie, some participants might have smoked hours before their visit and others might have smoked minutes before their visit), we deemed it reasonable to expect smokers who complied with the reduction schedule to have reduced their expired CO level by ≥50%. Other studies evaluating the use of precessation patches and denicotinized cigarettes for smoking cessation have adopted a similar approach of using the level of baseline CO reduction as a measure of treatment exposure and have noted a considerable treatment effect among those achieving >50% (55.6%) level of reduction [71].

Using this cutoff (≥50% CO reduction from baseline), we created subsets of participants within the SSNP and SS groups who were categorized as compliant (SSSP compliant and SS compliant) or noncompliant (SSNP noncompliant and SS noncompliant). These subsets were compared with the controls (EUC) in the secondary analyses described below in the Compliance Analysis section.

#### Statistical Analysis

The primary abstinence outcomes were CO-verified, self-reported, 7-day point prevalence abstinence at 2 and 4 weeks after the quit date. In addition, we examined self-reported 7-day point prevalence abstinence recorded at 6-month and 12-month follow-up visits. Logistic regression analysis was used to regress abstinence outcome in the treatment groups, which were initially unadjusted for any covariates. Additional multiple logistic regression analyses were conducted to adjust for the covariates of age, gender, race and ethnicity, and education. We report Bonferroni-adjusted *P* values calculated based on the number of comparisons within each analysis. For both the unadjusted and adjusted logistic regressions, our primary comparisons were between the 2 designated treatment groups (SSNP and SS) and the control group (EUC). Secondary analyses were also conducted using the 4 group subsets based on CO reduction–derived compliance (SSNP compliant, SSNP noncompliant, SS compliant, and SS noncompliant) versus control (EUC). In both the primary and secondary analyses, abstinence values were reported using both ITT and RO approaches to missing data. The statistical program Stata (version 15; StataCorp) was used for all analyses.

#### Compliance Analyses: Instrumental Variable Approach

Although our secondary analyses took scheduled smoking compliance into account when assembling the subgroups, we acknowledge that this type of analysis can be biased because noncompliance with the scheduled smoking cannot be assumed to be random with respect to abstinence, and therefore, confounding of abstinence by compliance may occur [72]. To account for this possible source of confounding, we complemented our primary abstinence outcome analyses by using an instrumental variable (IV) approach to estimate the actual causal effect of compliance on abstinence. This analytical approach allowed us to reduce bias in the outcome (abstinence) owing to differential participant compliance with schedule smoking. For this purpose, we conducted IV analysis [73] to estimate the local average treatment effect (LATE). The IV was the actual treatment indicator variable, which was directly related to compliance and only related to abstinence through compliance. This means that any effect of the scheduled smoking on abstinence was channeled through compliance. In an ITT analysis, where missing data on smoking status are imputed as “smoking,” the actual treatment effect can be decomposed into two parts: (1) the effect (abstinence rate) observed among those compliant with the treatment multiplied by the proportion of the sample that complied with the scheduled smoking and (2) the effect (abstinence rate) observed among those noncompliant with the scheduled smoking multiplied by the proportion of sample made up of noncompliers. Thus, the ITT abstinence rate can be expressed as follows: (proportion of compliers × effect of compliers) + (proportion of noncompliers × effect of noncompliers). By solving for the effect of compliance, we can identify and estimate the ITT abstinence owing to compliance (ITT [compliance] = (overall ITT abstinence rate) / proportion of the sample complying with treatment).

The IV analytical approach involved a 2-stage estimation of the treatment effects. In the first stage, we estimated the decomposition between the 2 error terms, accounting for unobserved confounding in the relationship between compliance and abstinence [74]. We estimated the LATE using the extended probit models [75] for the outcome using the “eprobit” function in Stata [76]. As the probit coefficients are not directly interpretable, we present the probabilities of abstinence of SSNP versus EUC and SS versus EUC estimated by using the “estatteffects” postestimation function, which produces the average treatment effect on the complier’s probability that they will comply with the treatment using actual treatment as the instrument (instrument defined as the variable that accounts for confounding). In the second stage, we estimated the average treatment effect on the treated using the predicted values of compliance derived from stage 1 estimation, rather than actual compliance [74], using full-information maximum likelihood. Full-information maximum likelihood considers the correlation between the error terms in the equation of the prediction of compliance and the error term in the prediction of abstinence.

### Ethical Considerations, Informed Consent, and Participation

Written informed consent was obtained from each participant at screening. All participants received a US $50 gift certificate for completion of visits through the end-of-treatment visit and a US $15 gift certificate for the completion of the follow-up visits. Participants also received a total of US $5 to US $50 gift certificates based on completion of ecological momentary assessments. This research was approved by MD Anderson’s institutional review board (PA15-0675).

## Results

### Sample Characteristics

**Figure 1:**
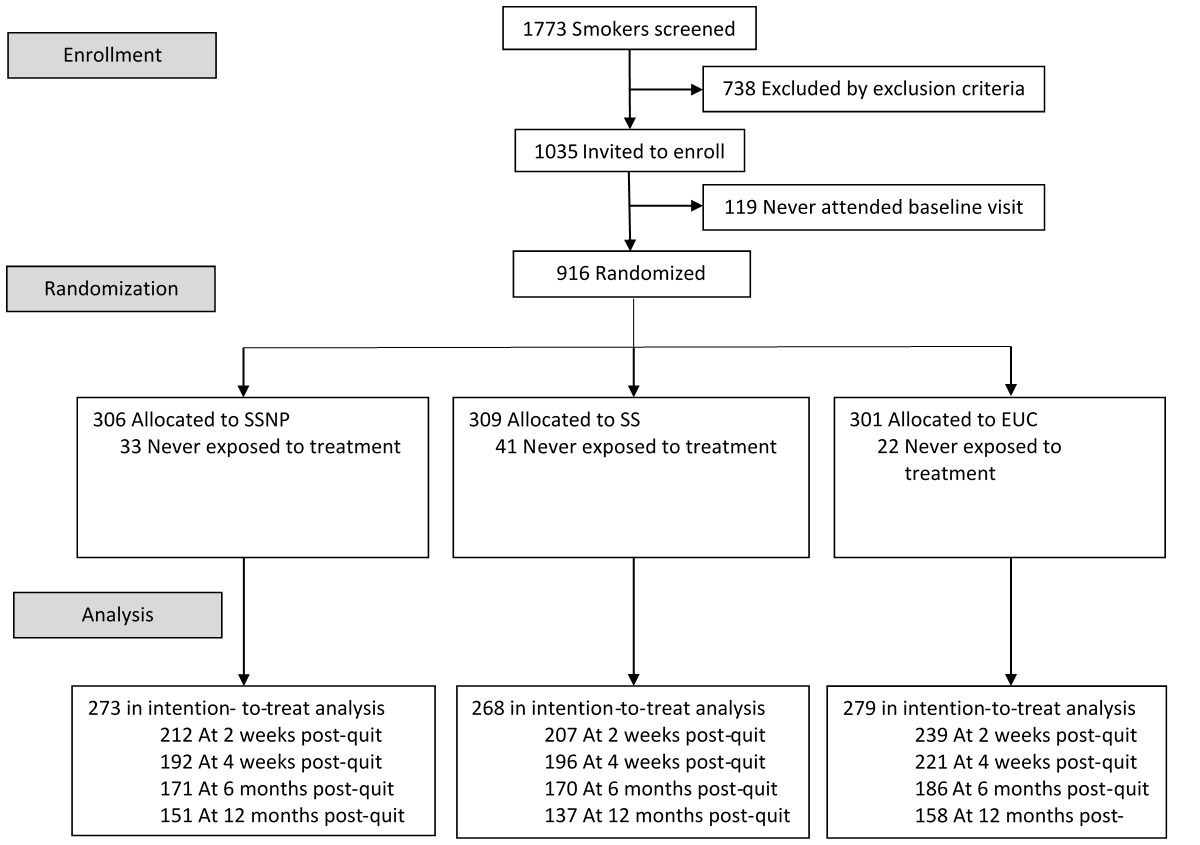
Consolidated Standards of Reporting Trials (CONSORT) diagram for patient allocation. EUC: enhanced usual care; SS: scheduled smoking only with no precessation patch; SSNP: scheduled smoking plus a precessation nicotine patch.

As shown in Figure 1, a total of 1773 smokers were screened for the study; 738 were excluded based on study exclusion criteria; 1035 were invited to enroll; and 916 presented at the baseline for randomization, with 306, 309, and 301 participants allocated to the SSNP, SS, and EUC groups, respectively. However, 33, 41, and 22 participants in each of the respective groups returned their HD unused and were excluded from the analysis for not having been exposed to the intervention.

The baseline demographic characteristics for the 3 main treatment groups (SSNP, SS, and EUC) and the 4 compliance groups (SSNP compliant, SSNP noncompliant, SS compliant, and SS noncompliant) are presented in Table 1. Typical for smoking cessation trials, participants had an average age of approximately 43 (SD 11.02) years, were mostly White, were equally distributed between male and female, smoked slightly more than a pack of cigarette per day (mean 23, SD 10.33), had been smoking for an average of approximately 24 years, and were moderately dependent on nicotine (mean Fagerström Test for Cigarette Dependence score 5, SD 1.98). The Center for Epidemiologic Studies Depression Scale depression total score and the Positive and Negative Affect Scale scores were consistent across the 3 treatment groups. No significant differences were found between the treatment and EUC groups on any of these baseline characteristics.

**Table 1:** Demographic characteristics at baseline across the 3 main treatment groups (scheduled smoking plus a precessation nicotine patch [SSNP], scheduled smoking only with no precessation patch [SS], and enhanced usual care [EUC]) as well as the 4 compliance groups (SSNP compliant, SSNP noncompliant, SS compliant, and SS noncompliant by ≥50% reduction in carbon monoxide criteria).

Characteristics	Randomized groups					Compliance groups				
	EUC (n=279)	SS (n=268)	SSNP (n=273)	*P* value	SS noncompliant (n=197)		SSNP noncompliant (n=134)	SS compliant (n=71)	SSNP compliant (n=139)	*P* value
Age (years), mean (SD)	42.18 (10.9)	42.51 (11.22)	43.23 (10.94)	.52	43.12 (11.41)		43.95 (11.26)	40.83 (10.58)	42.54 (10.62)	.32
Female, n (%)	140 (50.2)	134 (50)	131 (48)	.85	101 (51.3)		66 (49.3)	33 (46.5)	65 (46.8)	.91
African American, n (%)	58 (20.8)	47 (17.5)	40 (14.7)	.09	38 (19.3)		27 (20.1)	9 (12.7)	13 (9.4)	.05
European American, n (%)	188 (67.4)	201 (75)	208 (76.2)	.09	144 (73.1)		94 (70.1)	57 (80.3)	114 (82)	.05
Hispanic or other, n (%)	33 (11.8)	19 (7.1)	25 (9.2)	.09	14 (7.1)		13 (9.7)	5 (7)	12 (8.6)	.05
Less than or equal to high school, n (%)	49 (17.6)	65 (24.3)	53 (19.4)	.34	45 (22.8)		24 (17.9)	20 (28.2)	29 (20.9)	.51
Some college, n (%)	137 (49.1)	114 (42.5)	127 (46.5)	.34	83 (42.1)		60 (44.8)	31 (43.7)	67 (48.2)	.51
Greater than or equal to college graduate, n (%)	93 (33.3)	89 (33.2)	93 (34.1)	.34	69 (35)		50 (37.3)	20 (28.2)	43 (30.9)	.51
CES-D^a^ total score, mean (SD)	9.64 (8.25)	9.02 (6.86)	9.09 (7.89)	.59	9.26 (6.82)		10.36 (8.22)	8.34 (6.95)	7.86 (7.38)	.06
PANAS^b^ negative affect, mean (SD)	34.05 (7.79)	34.09 (7.20)	35.00 (7.51)	.26	34.37 (7.24)		35.22 (7.79)	33.33 (7.08)	34.78 (7.25)	.41
PANAS positive affect, mean (SD)	18.16 (7.30)	17.80 (6.42)	17.37 (5.90)	.37	18.15 (6.53)		17.41 (5.62)	16.86 (6.06)	17.32 (6.19)	.41
CPD^c^, mean (SD)	23.36 (9.35)	23.38 (10.16)	23.33 (9.21)	.99	23.70 (10.86)		24.07 (10.28)	22.52 (7.88)	22.61 (8.01)	.67
FTND^d^, mean (SD)	5.02 (1.98)	5.05 (2.01)	5.12 (1.96)	.83	5.01 (2.00)		5.26 (2.04)	5.16 (2.05)	4.99 (1.89)	.76
Years smoking, mean (SD)	23.07 (10.9)	23.69 (11.19)	24.88 (10.90)	.15	24.26 (11.35)		25.51 (11.06)	22.10 (10.65)	24.27 (10.76)	.15

^a^CES-D: Center for Epidemiologic Studies Depression Scale.

^b^PANAS: Positive and Negative Affect Scale.

^c^CPD: cigarettes smoked per day.

^d^FTND: Fagerström Test for Nicotine Dependence.

### Smoking Reduction Compliance

Collectively, participants in the SSNP and SS groups did not differ from each other in the percentage of scheduled cigarettes smoked (SSNP: 76% and SS: 74%; *F*_1,487_=0.61; *P*=.43) or the number of off-scheduled cigarettes smoked (SSNP: 24 and SS: 25; *F*_1,487_=0.31; *P*=.58) during the 3 precessation week (reduction) period.

Significantly more participants from the SSNP group than those in the SS group achieved ≥50% reduction in expired CO (Wald *X*^2^_521_=5.5; *P*=.02) during the precessation phase. Specifically, 50.9% (139/273) of all participants assigned to the SSNP group achieved ≥50% reduction in expired CO level between their baseline and last precessation visit, whereas only 26.5% (71/268) of those assigned to the SS group reduced their expired CO level by ≥50%. Smokers in the SSNP group who achieved ≥50% CO reduction were more likely than those who failed to reduce their CO by 50% to have smoked their scheduled cigarettes on time (SSNP compliant: 80% and SSNP noncompliant: 71%; *t*_1,521_=5.34; *P*<.001). However, the 2 groups did not differ in the number of cigarettes smoked off schedule (unscheduled cigarettes) during the 3-week precessation period (SSNP compliant: 26 and SSNP noncompliant: 22; *t*_1,521_=0.978; *P*=.33). Similarly, smokers in the SS group who reduced their CO by ≥50% were more likely than those in the SS group who failed to reduce their CO by 50% to smoke on schedule (SS compliant: 78% and SS noncompliant: 72%; t_903_=2.56; *P*=.01). The 2 groups did not differ in the number of unscheduled cigarettes smoked during the 3-week precessation period (SS compliant: 25 and SS noncompliant: 25; t_903_=0.09; *P*=.92).

### Nicotine Patch Compliance

Participants assigned to the SSNP group reported a high rate of compliance to precessation use of the nicotine patch, applying an average of 97% (SD 10.7%) of all patches as instructed before their target quit date. Prequit nicotine patch compliance for SSNP compliant was 97% and for SSNP noncompliant was 95%. Postquit nicotine patch use was also assessed, and we found no significant difference in compliance across all groups at 2 weeks (SSNP: 90.96%, SS: 89.25%, and EUC: 90.72%; *F*_2,626_=0.37; *P*=.69) and 4 weeks (SSNP: 81.88%, SS: 82.22%, and EUC: 80.20%; *F*_2,566_=0.22; *P*=.80) after the target quit date. Across the 4 compliance groups, use of nicotine patch was also similar with no significant differences for the 2 weeks postquit (SSNP compliant: 91.43%, SS compliant: 92.62%, SSNP noncompliant: 89.56%, and SS noncompliant: 87.4%; *F*_3,504_=0.4; *P*=.75) as well as for the 4 weeks postquit time points (SSNP compliant: 86.10%, SS compliant: 83.01%, SSNP noncompliant: 73.81%, and SS noncompliant: 81.57%; *F*_3,367_=1.93; *P*=.12).

From a design perspective, this high level of patch compliance means that after the quit date, the 3 treatment groups (SSNP, SS, and EUC) were essentially the same, that is, scheduled smoking was completed, and all were now equally exposed to a standard regimen of NRT.

### Abstinence Outcome

#### Treatment Groups Without Regard to Smoking Reduction Compliance

**Figure 2:**
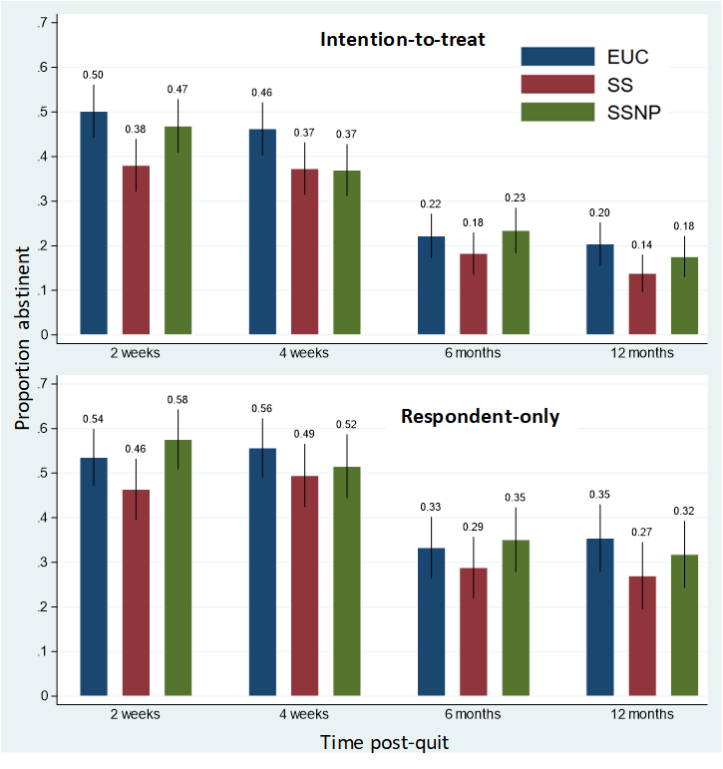
Abstinence rates with 95% CIs for all 4 time points by intention-to-treat and respondent-only analyses and by treatment group. Note: Response rates for enhanced usual care (EUC): 239 At 2 weeks post-quit, 221 at 4 weeks post-quit, 186 at 6 months post-quit and 158 at 12 months post-quit; for scheduled smoking only with no precessation patch (SS): 207 at 2 weeks post-quit, 196 at 4 weeks post-quit, 170 at 6 months post-quit, 137 at 12 months post-quit; for scheduled smoking plus a precessation nicotine patch (SSNP): 212 at 2 weeks post-quit, 192 at 4 weeks post-quit, 171 at 6 months post-quit, 151 at 12 months post-quit. Patch compliance: 2 weeks post-quit (SSNP: 90.96%, SS: 89.25%, EUC: 90.72%); 4 weeks post-quit (SSNP: 81.88%, SS: 82.22%, EUC: 80.20%) after the target quit date.

**Figure 3:**
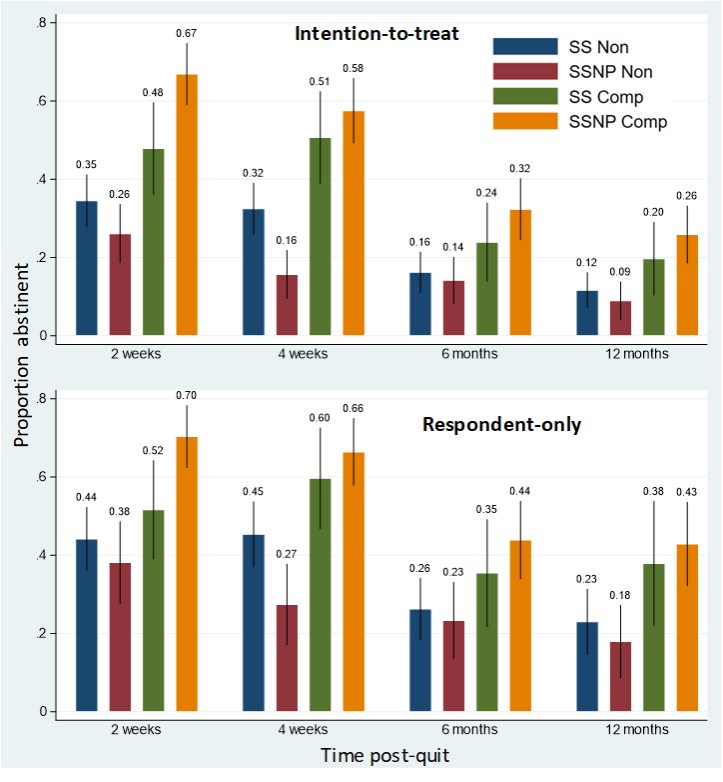
Abstinence rates with 95% CIs for all 4 time points by intention-to-treat and respondent-only analyses and by scheduled smoking compliance groups. Note: Patch compliance: 2 weeks post-quit: scheduled smoking plus a precessation nicotine patch compliant (SSNP Comp): 91.43%, scheduled smoking only with no precessation patch compliant (SS Comp): 92.62%, scheduled smoking plus a precessation nicotine patch non-compliant (SSNP Non): 89.56%, scheduled smoking only with no precessation patch non-compliant (SS Non): 87.4%; 4 weeks post-quit time: SSNP Comp: 86.10%, SS Comp: 83.01%, SSNP Non: 73.81%, SS Non: 81.57%.

Our primary abstinence outcome (7-day point prevalence abstinence) was examined at 2 weeks, 4 weeks (primary), 6 months, and 12 months after the target quit date. Overall abstinence rates for the 3 main treatment groups (SSNP, SS, and EUC) and 4 compliance groups are presented in Figures 2 and 3, respectively. Logistic regression analyses were conducted to evaluate whether scheduled smoking with (SSNP) or without (SS) the precessation transdermal nicotine patch (SSNP) was superior to the control group (EUC) in helping smokers quit. As shown in Table 2, no significant differences between SSNP and EUC or SSNP and SS were found on abstinence at any of the 4 time points, in both the adjusted and unadjusted models, for both ITT and RO abstinence outcomes approach. However, in the ITT model only, both adjusted and unadjusted models indicated a lower probability of abstinence for SS versus EUC at week 2 only. No significant differences were observed at any of the other postcessation time points.

**Table 2:** Effects of treatment on respondent-only and intention-to-treat abstinence for all 4 time points for the 3 main treatment groups (scheduled smoking plus a precessation nicotine patch [SSNP], scheduled smoking only with no precessation patch [SS], and enhanced usual care [EUC]).

				2 weeks after the quit date					4 weeks after the quit date					6 months after the quit date					12 months after the quit date		
				OR^a^ (95% CI)		*P* value^b^		OR (95% CI)			*P* value^b^		OR (95% CI)			*P* value^b^		OR (95% CI)			*P* value^b^
**Intenti** **on** **-to-treat**																					
	**Unadjusted**																				
		SSNP versus EUC	0.88 (0.63-1.22)		>.99		0.68 (0.49-0.96)			.08		1.07 (0.72-1.60)			>.99		0.83 (0.54-1.27)			>.99	
		SS versus EUC	0.61 (0.43-0.86)		.01		0.69 (0.49-0.97)			.10		0.78 (0.52-1.19)			.75		0.62 (0.40-0.98)			.12	
		SSNP versus SS	1.44 (1.02-2.02)		.11		0.99 (0.70-1.40)			>.99		1.37 (0.90-2.08)			.42		1.33 (0.84-2.12)			.68	
	**Adjusted^c^**																				
		SSNP versus EUC	0.87 (0.62-1.22)		>.99		0.67 (0.48-0.95)			.07		1.09 (0.73-1.63)			>.99		0.83 (0.54-1.28)			>.99	
		SS versus EUC	0.62 (0.44-0.87)		.02		0.70 (0.49-0.98)			.13		0.80 (0.53-1.22)			.79		0.63 (0.40-1.00)			.12	
		SSNP versus SS	1.41 (1.00-1.99)		.16		0.97 (0.68-1.37)			>.99		1.36 (0.89-2.07)			.41		1.32 (0.83-2.11)			.65	
**Respondent only**																					
	**Unadjusted**																				
		SSNP versus EUC	1.11 (0.76-1.61)		>.99		0.83 (0.56-1.21)			.99		1.20 (0.78-1.85)			>.99		0.86 (0.54-1.37)			>.99	
		SS versus EUC	0.70 (0.48-1.01)		.16		0.78 (0.53-1.14)			.58		0.83 (0.53-1.30)			>.99		0.67 (0.41-1.11)			.34	
		SSNP versus SS	1.59 (1.08-2.34)		.05		1.06 (0.72-1.58)			>.99		1.45 (0.92-2.28)			.32		1.27 (0.77-2.12)			>.99	
	**Adjusted** ^c^																				
		SSNP versus EUC	1.12 (0.76-1.63)		>.99		0.81 (0.55-1.20)			.91		1.26 (0.81-1.95)			>.99		0.92 (0.57-1.49)			>.99	
		SS versus EUC	0.71 (0.49-1.03)		.23		0.77 (0.53-1.14)			.62		0.86 (0.55-1.35)			>.99		0.70 (0.42-1.15)			.35	
		SSNP versus SS	1.58 (1.07-2.32)		.07		1.05 (0.70-1.57)			>.99		1.46 (0.93-2.31)			.28		1.32 (0.79-2.22)			.97	

^a^OR: odds ratio.

^b^*P* values are corrected for multiple comparisons (Bonferroni).

^c^Models were adjusted for age, sex, race, and education.

#### Treatment Groups With Regard to Smoking Reduction Compliance (As Treated)

Although no main effects of SSNP versus EUC group were observed in relation to the smoking cessation outcome, this could partially be owing to varying degrees of compliance with the scheduled smoking intervention and its downstream effects on the actual reduction in smoke exposure. To account for the effect of smoking reduction compliance on treatment efficacy, as noted above in the section Compliance Analysis, we used a ≥50% reduction in expired CO as a cutoff criterion to subdivide each of the 2 treatment groups into either compliant (SSNP compliant and SS compliant) or noncompliant groups (SSNP noncompliant and SS noncompliant).

As shown in Table 3, the results of the logistic regression showed that participants in the SSNP-compliant group were significantly more likely to report 7-day ITT abstinence than those in the EUC group at 2 weeks (odds ratio [OR] 2.01, 95% CI 1.31-3.07), 4 weeks (OR 1.58, 95% CI 1.05-2.38), and 6 months (OR 1.68, 95% CI 1.04-2.64) after the scheduled quit date, although the *P* values for the 4-week and 6-month comparisons were attenuated following the Bonferroni adjustment for 10 comparisons. No significant difference in abstinence was found between the SSNP-compliant and EUC groups 12 months after cessation. The adjusted and RO results reflected the same findings, with the *P* value of 0.04 for the 2-week SSNP compliant versus EUC comparison remaining significant after correction. Both the SSNP-noncompliant and SS-noncompliant groups were significantly less likely to abstain than EUC at 2 weeks, 4 weeks, and 12 months postcessation in the adjusted and unadjusted ITT models but not in the RO models.

SSNP-compliant participants were also more likely than SS-compliant participants to report 7-day abstinence at 2 weeks after the quit date (OR 2.20, 95% CI 1.23-3.95) in the ITT unadjusted comparison, with similar findings for the adjusted ITT and RO analyses, although these later comparisons did not survive the Bonferroni-adjusted correction. Although SSNP-compliant smokers continued to outperform SS-compliant smokers in the 7-day abstinence at 4 weeks, 6 months, and 12 months after cessation, the differences were not statistically significant. No other significant differences in abstinence outcomes were found between SSNP-compliant and SS-compliant participants at either 6 or 12 months after cessation.

**Table 3:** Effects of treatment compliance groups on respondent-only and intention-to-treat (ITT) abstinence for all 4 time points for the 4 compliance groups (scheduled smoking plus a precessation nicotine patch [SSNP] compliant, SSNP noncompliant, scheduled smoking only with no precessation patch [SS] compliant, and SS noncompliant by ≥50% reduction in carbon monoxide criteria). EUC: enhanced usual care.

				2 weeks after the quit date				4 weeks after the quit date				6 months after the quit date					12 months after the quit date						
				OR^a^ (95% CI)		*P* value		OR (95% CI)		*P* value		OR (95% CI)		*P* value		OR (95% CI)			*P* value				
**ITT**																							
	**Unadjusted**																						
		SSNP compliant versus EUC	2.01 (1.31-3.07)		.008		1.58 (1.05-2.38)		.28		1.68 (1.06-2.64)		.30		1.36 (0.84-2.20)			>.99					
		SSNP noncompliant versus EUC	0.35 (0.22-0.55)		<.001		0.22 (0.13-0.36)		<.001		0.58 (0.33-1.01)		.40		0.38 (0.20-0.74)			.009					
		SS compliant versus EUC	0.91 (0.54-1.54)		>.99		1.20 (0.71-2.01)		>.99		1.10 (0.60-2.04)		>.99		0.96 (0.50-1.84)			>.99					
		SS noncompliant versus EUC	0.52 (0.36-0.76)		.005		0.56 (0.38-0.82)		.02		0.68 (0.42-1.09)		.99		0.51 (0.31-0.87)			.09					
		SSNP compliant versus SS compliant	2.20 (1.23-3.95)		.08		1.32 (0.74-2.34)		>.99		1.52 (0.79-2.91)		>.99		1.42 (0.71-2.86)			>.99					
	**Adjusted**																						
		SSNP compliant versus EUC	2.03 (1.32-3.13)		.007		1.59 (1.05-2.41)		.26		1.77 (1.12-2.80)		.26		1.43 (0.88-2.33)			>.99					
		SSNP noncompliant versus EUC	0.34 (0.22-0.54)		<.001		0.21 (0.12-0.35)		<.001		0.57 (0.33-1.00)		.34		0.37 (0.19-0.72)			.006					
		SS compliant versus EUC	0.95 (0.56-1.60)		>.99		1.25 (0.74-2.11)		>.99		1.18 (0.63-2.18)		>.99		1.02 (0.53-1.98)			>.99					
		SS noncompliant versus EUC	0.53 (0.36-0.77)		.008		0.56 (0.38-0.82)		.02		0.69 (0.43-1.11)		.98		0.51 (0.30-0.87)			.07					
		SSNP compliant versus SS compliant	2.15 (1.19-3.87)		.11		1.28 (0.72-2.27)		>.99		1.51 (0.78-2.89)		>.99		1.40 (0.69-2.82)			>.99					
**Respondent only**																							
	**Unadjusted**																						
		SSNP compliant versus EUC	1.87 (1.18-2.95)		.05		1.54 (0.97-2.45)		.61		1.69 (1.03-2.78)		.40		1.39 (0.81-2.39)			>.99					
		SSNP noncompliant versus EUC	0.53 (0.32-0.88)		.12		0.30 (0.17-0.53)		<.001		0.71 (0.39-1.30)		>.99		0.40 (0.20-0.80)			.04					
		SS compliant versus EUC	0.87 (0.50-1.52)		>.99		1.23 (0.68-2.22)		>.99		1.13 (0.58-2.20)		>.99		1.08 (0.52-2.26)			>.99					
		SS noncompliant versus EUC	0.63 (0.42-0.95)		.27		0.64 (0.42-0.98)		.39		0.73 (0.44-1.20)		>.99		0.55 (0.31-0.97)			.29					
		SSNP compliant versus SS compliant	2.14 (1.15-4.01)		.18		1.25 (0.65-2.41)		>.99		1.49 (0.73-3.04)		>.99		1.29 (0.58-2.83)			>.99					
	**Adjusted**																						
		SSNP compliant versus EUC	1.90 (1.19-3.03)		.04		1.53 (0.95-2.44)		.64		1.83 (1.10-3.03)		.34		1.59 (0.91-2.78)			>.99					
		SSNP noncompliant versus EUC	0.54 (0.32-0.90)		.16		0.29 (0.17-0.52)		<.001		0.72 (0.39-1.33)		>.99		0.42 (0.20-0.84)			.03					
		SS compliant versus EUC	0.91 (0.52-1.60)		>.99		1.26 (0.69-2.28)		>.99		1.19 (0.61-2.34)		>.99		1.23 (0.58-2.60)			>.99					
		SS noncompliant versus EUC	0.65 (0.43-0.98)		.38		0.64 (0.42-0.98)		.42		0.75 (0.45-1.25)		>.99		0.55 (0.31-0.99)			.28					
		SSNP compliant versus SS compliant	2.09 (1.11-3.92)		.24		1.21 (0.63-2.34)		>.99		1.53 (0.75-3.13)		>.99		1.29 (0.58-2.87)			>.99					

^a^OR: odds ratio.

#### Causal Effect Estimation Through LATE

We complemented the ITT and as-treated analyses with the LATE method using the IV analysis. In the LATE analyses (Table 4), we examined the effect of complying with the intervention for each of the 3 groups and found a substantial difference in abstinence rates at 2 weeks after cessation between the SSNP and EUC groups (difference=26%, 95% CI 18%-34%) and at the 4-week time point (difference=29%, 95% CI 20%-37%). Significant results were also identified when comparing compliers of SS with EUC at week 2 (difference=27%, 95% CI 19%-36%) and week 4 (difference=33%, 95% CI 24%-41%). The results of the RO analysis were consistent with the ITT results despite the narrower differences between treatment groups. Overall, the LATE analysis using the IV method showed that complying with the SSNP or the SS intervention resulted in at least a 19% difference in abstinence rates compared with the control group at 2 and 4 weeks after cessation, which was the primary abstinence outcome.

**Table 4:** Average causal effect on the treated from local average treatment effect analysis.

		2 weeks after the quit date		4 weeks after the quit date			6 months after the quit date			12 months after the quit date	
		Difference (95% CI)	*P* value	Difference (95% CI)	*P* value	Difference (95% CI)		*P* value	Difference (95% CI)		*P* value
**Intention-to-treat**											
	SSNP^a^ versus EUC^b^	0.26 (0.18 to 0.34)	<.001	0.29 (0.20 to 0.37)	<.001	0.26 (0.01 to 0.51)		.24	0.22 (−0.21 to 0.77)		.26
	SS^c^ versus EUC	0.27 (0.19 to 0.36)	<.001	0.33 (0.24 to 0.41)	<.001	0.29 (−19 to 0.78)		.34	0.28 (−0.03 to 0.47)		.09
**Respondent only**											
	SSNP versus EUC	0.19 (0.10 to 0.25)	<.001	0.18 (0.10 to 0.26)	<.001	0.29 (−0.02 to 0.60)		.07	0.01 (−0.33 to 0.34)		.97
	SS versus EUC	0.19 (0.11 to 0.27)	<.001	0.21 (0.13 to 0.29)	<.001	0.34 (−0.31 to 1.00)		.31	−0.12 (−0.60 to 0.35)		.60

^a^SSNP: scheduled smoking plus a precessation nicotine patch.

^b^EUC: enhanced usual care.

^c^SS: scheduled smoking only with no precessation patch.

#### Effects of Treatment on Withdrawal and Affect

We modeled withdrawal and affect outcomes using the flexible mixed effects model framework to account for repeated measures, including the 2- and 4-week and 6- and 12-month time points, using the “xtmixed” command in Stata [76]. The time-dependent direct effects of treatment status on the outcomes as well as the difference in the rate of change in outcomes between treatment groups during the duration of the study were the main effects of interest. We tested the hypothesis that there would be no difference in the rate of change among the EUC, SS, and SSNP groups by including a treatment-by-time interaction in the models. The reference category for all models was the EUC group. All models were estimated using maximum likelihood, which provides similar results to multiple imputation when the missingness is assumed to be missing at random [77]. Results of this analyses are presented in Table S1 in Multimedia Appendix 1.

## Discussion

### Principal Findings

This innovative clinical trial evaluated the effects of a mobile smoking cessation intervention designed to progressively reduce smoking before quitting using SSNP, in comparison with SS and an EUC control group. EUC smokers used the nicotine patch on the quit date and carried the same mobile device to record cigarettes and manage their quit attempt, with no scheduled reduction. No counseling was provided in this study beyond the explanation of the use and rationale of the procedures and an electronic tip guide available on the mobile device. The results showed no overall differences in abstinence in the 3 groups over multiple time points (2 weeks, 4 weeks, 6 months, and 12 months posttarget quit date) in both unadjusted and adjusted (for covariates) analyses using both ITT (missing smoking status imputed as smoking) and RO (no imputation) approaches to the treatment of missing data. However, when taking schedule compliance into account, a different pattern of results emerged.

We evaluated schedule compliance in 2 types of analyses, and the results in both were consistent, showing that smokers who were compliant with the scheduled smoking procedure and used the nicotine patch before the quit date were more likely to be abstinent at several time points, with the differences at 2 weeks and 4 week after quitting (primary outcome of the study) being the most robust.

Specifically, when we subdivided the 2 scheduled smoking groups into those who complied with the scheduled reduction, as defined by a 50% drop in expired CO between baseline and final week of scheduled smoking, and those who did not, we found that the SSNP compliers abstained significantly more often at the 2-week, 4-week, and 6-month postquit dates when compared with the EUC group. Although the differences at 4 weeks and 6 months were not significant following the correction for multiple comparisons, they continued to trend in the correct direction, with ORs >1.5, which have generally been considered as a clinically meaningful difference between treatments.

We also took an IV approach to evaluate compliance, using LATE estimates of the direct *causal* effect of the smoking cessation intervention among compliers. In comparison with the EUC group, those who complied with the intervention in the SSNP group averaged a 26%, 29%, and 22% increase in abstinence at the 2-week, 4-week, and 6-month postquit time points, respectively, although the 6-month outcome was not statistically different. Thus, this LATE analysis offers support for the value of the SSNP intervention when compliance is considered. The LATE analysis complements the standard effectiveness analyses because the LATE analysis evaluates the effect of actually receiving the intervention. We found no differences across the 3 groups in postquit compliance with patch use. However, precessation patch use was related to schedule compliance, as noted by the fact that >50% of the smokers in the SSNP group achieved a >50% reduction in CO (a criterion of compliance) compared with 26% in the SS group. However, both the direction and the magnitude of the effects of SSNP schedule compliance in both compliance analyses gives us confidence that compliance with the scheduled smoking procedure is a key factor for the intervention to work. This conclusion is further supported by the absence of precessation differences in compliance with the nicotine patch between the 2 SSNP compliance groups (SSNP compliant: 97% and SSNP noncompliant: 95%). Thus, although use of the nicotine patch before cessation may make it easier for smokers to comply with the scheduled reduction procedure, compliance with precessation patch use alone is insufficient to improve smoking cessation outcomes over usual care. Moreover, we noted that the SSNP intervention had other beneficial effects on the quitting experience, such as reducing withdrawal, negative affect, and craving, which could reduce the probability of relapse, and may encourage future quit attempts should that occur, particularly because we know that it may take multiple quit attempts before a smoker eventually succeeds [8]. Subsequent studies should evaluate ways to promote compliance, notably through smoking cessation counseling, which was not conducted in this study.

As noted earlier, previous studies on gradual reduction methods have been largely inconclusive when compared with abrupt quitting (usual care); however, the efficacy of precessation nicotine patch use has received modest support. Our results suggest that the combined use of nicotine patch and scheduled smoking (a method of gradual reduction) can be effective when compliance with the scheduled smoking procedure is observed. This is consistent with a study by Rose et al [71] that showed that the beneficial effects of precessation patch use on smoking cessation are strongly related to the extent of precessation smoking reduction as measured by changes in expired CO from baseline to the quit date, which we used as a criterion for assessing schedule compliance. Our results are also consistent with previous studies suggesting that scheduled smoking has positive effects on the quitting experience, reducing withdrawal, negative affect, and craving.

### Limitations

Although the overall comparisons showed no differences across the groups, there are limitations in the study design that should be noted. First, the main component of this intervention was implemented before the quit date and involved simultaneous use of both the nicotine patch and the scheduled smoking procedure. However, the postquit intervention was essentially the same for all groups and consisted of continued use of the HD for an additional 2 weeks plus use of the nicotine patch. Thus, the expectation that a 3-week prequit intervention might have long-lasting effects on postquitting behavior would require a very large precessation treatment effect, when averaging across all participants within a group. Our compliance analyses suggest that the effects of the combined scheduled smoking and nicotine patch can persist into the postquitting environment, with clinically meaningful effects up to 6 months after quitting among smokers who adhere to the procedure. This is analogous to the notion that a drug cannot work if it is not taken, and a key factor is to determine why it is not used and to intervene if possible. This intervention by design did not involve smoking cessation counseling, which we know is essential to produce improved outcomes when combined with pharmacotherapy [35]. Although we envisioned an intervention that would function autonomously without counseling, the program could have been enhanced by incorporating modern text messaging, chatbots, or artificial intelligence components to monitor and enhance compliance as well as provide limited motivational counseling. These features are incorporated into our current scheduled smoking program. Moreover, providing the SSNP intervention in the context of traditional smoking cessation counseling may be an important implementation strategy. Such an approach could easily be incorporated into state quitline interventions that involve text message interventions. Quitline counseling could provide counseling and monitor adherence.

Another limitation was the provision of HD in the control group. This was done to achieve internal validity, that is, all smokers carried a device that delivered the scheduled smoking intervention to both scheduled groups in the precessation period and functioned essentially the same for all smokers in the postquit environment. Thus, the control group had access to the relapse management routines, tip guides, and self-assessments, all of which might have had an impact on postquit date smoking behavior and raising cessation rates beyond what might be expected through usual care (ie, quitting abruptly, with the nicotine patch). None of these features would normally be available under true usual care control groups; hence, this group was designated as “Enhanced Usual Care.”

This study evaluated the combination of a low-touch behavioral treatment (scheduled smoking) delivered on a HD with and without NRT against a usual care NRT control condition. It could be argued that because our findings and program used to produce the data were >20 years old, they may have limited applicability to the current treatment standards, technology, and the current population of smokers. However, we believe that our findings are highly relevant to the current smoking cessation treatment for several reasons. First, the basic standard of care for smoking cessation till date is NRT, typically using the nicotine patch [10]. It is by far the most used pharmacotherapy with quitlines [36] and low-touch interventions such this study. In addition, as described earlier, gradual reduction methods remain attractive to many smokers trying to quit; hence, both the behavioral (scheduled smoking) and pharmacological (NRT) treatments fit into today’s approaches to smoking cessation. Second, although smoking rates have declined nationally from approximately 23% in 2002 [37] to 12.5% in 2020 [38], our inclusion criteria for this study included smoking ≥10 cigarettes smoked per day at baseline, which captures most smokers (approximately 75% in 2015 [39]—latest available data). Third, with regard to the technology used to deliver the intervention, the hardware device is obsolete, but as indicated earlier, we have adapted (and enhanced) each of the elements of the program for use on currently available smartphones (Android) and have been testing the program in a clinical setting. We intend to make the program available to the research community and include an iOS version when complete.

### Conclusions

Scheduled smoking, when combined with precessation use of NRT, can result in significantly higher abstinence rates than usual care (abrupt cessation with NRT), particularly in the early postquit phase (2 and 4 weeks after cessation) when smokers are compliant with the procedure. There was also evidence for a clinically meaningful effect (OR>1.5) at 6 months after quitting, although statistical differences were reduced with adjustments for multiple comparisons. Scheduled smoking also produces a better overall quitting experience by reducing symptoms of nicotine withdrawal and craving in comparison with usual care, which could encourage more future quit attempts. Future studies in this area should focus on the use of counseling or other methods to improve adherence.

## Appendix

Multimedia Appendix 1 Details on study design, intervention characteristics, and additional analyses.

## Abbreviations

CO: carbon monoxide
EUC: enhanced usual care
HD: handheld device
ITT: intention-to-treat
IV: instrumental variable
LATE: local average treatment effect
NRT: nicotine replacement therapy
OR: odds ratio
PRIME-MD: Primary Care Evaluation of Mental Disorders
RO: respondent-only
SS: scheduled smoking only with no precessation patch
SSNP: scheduled smoking plus a precessation nicotine patch

## Data Availability

The data supporting the findings of this study are available from the corresponding author upon reasonable request.
