# Supplementary material for: The Effects of Scheduled Smoking Reduction and Precessation Nicotine Replacement Therapy on Smoking Cessation: Randomized Controlled Trial With Compliance
Source: JMIR Form Res. 2023 Jun 20;7:e39487. doi: 10.2196/39487 (PMC10337448; doi:10.2196/39487)
Supplement: Multimedia Appendix 1 [file formative_v7i1e39487_app1.docx]

### Multimedia Appendix 1: Details on study design, intervention characteristics, and additional analyses.

### Introduction

Smoking remains a significant public health problem and is currently responsible for almost 6 million premature deaths each year worldwide, and as many as 8 million deaths annually by the year 2030 ^1^. Tobacco plays a causal role in at least 15 types of cancer ^2,3^, accounts for approximately 30% of the attributable risk for overall cancer mortality, and is responsible for over 87% of all lung cancer deaths, 61% of chronic obstructive lung disease deaths, and 32% of heart disease deaths. In the U.S., cigarette smoking accounts for as much as $333 billion dollars annually, including up to $176 billion in direct medical care costs and $151 billion in lost productivity from premature death ^4^.

Almost 70% of smokers report that they want to quit smoking every year ^5^, and while 55% report having made a serious attempt, annually, only ~8% achieve some level of success ^6^. In fact, the annual success rate of any one individual attempt at quitting on one’s own (without treatment) is 5% or less ^7^. Subsequent quit attempts after an initial try are extremely common, and for many smokers, multiple attempts are essential to achieve long-term success ^8^. The overall impact of successful smoking cessation on population health is substantial, including reducing the prevalence, morbidity, and/or mortality of 15 tobacco-related cancers, stroke, cardiovascular disease, diabetes, COPD, and rheumatoid arthritis, as well as associated healthcare expenditures ^4,9^. It is important to provide a variety of efficacious and appealing quitting options to encourage smokers, especially those who have tried to quit repeatedly without success, to make another quit attempt.

### Smoking Reduction/ Scheduled Smoking

An assortment of pharmacological and behavioral treatment strategies have been shown to increase cessation rates in adult smokers ^10-12^. Traditionally, formal guidelines primarily recommend that people stop smoking abruptly on a future (pre-specified) quit day, but smoke normally up until this day ^10,13^. An alternative method is to gradually reduce the amount of tobacco smoked (usually over the course of a few weeks) before quitting completely on the quit day. Gradual reduction methods have been implemented in several ways, such as limiting the time periods (or situations) in the day when smoking occurs (“smoke-free periods”) ^14^, using NRT or other types of pharmacotherapy to replace or help cope with cigarettes not smoked ^15,16^, setting goals to reduce by a certain number or percentage of cigarettes each day ^17^, or reducing smoking before quitting without a specific plan ^18^.

Some proponents of abrupt quitting have suggested that gradual reduction is not ideal because the cigarettes that are smoked may become more rewarding as a smoker cuts down, possibly leading to a decrease in the motivation or ability to quit completely ^19,20^. Research suggests, however, that the use of medications known to reduce withdrawal symptom severity, such as nicotine replacement therapy (NRT) or varenicline, may mitigate this concern, as they have been effective in smokers attempting to reduce smoking and/or reduce-to-quit smoking ^16,21^. Gradual reduction techniques may confer benefit to smokers in a variety of ways, such as increasing self-efficacy to quit by successfully completing interim goals (cutting down), slowly reducing the intensity of craving and other withdrawal symptoms as less nicotine is consumed through the process of cutting down, or weakening the link between smoking triggers and smoking behavior as less cigarettes are consumed ^19,22^. Survey studies suggest that gradual reduction is an appealing option to a substantial number of smokers ^23-25^ and may be particularly enticing to those who have previously failed to quit using abrupt methods, or have had more difficulty quitting in general. In addition, research suggests that smokers may naturally switch between abrupt strategies and gradual reduction strategies when attempting to modify their smoking behavior, likely not viewing the two options as mutually exclusive ^26^.

Comparisons between abrupt and gradual cessation methods has been largely inconclusive, likely due in part to the considerable variation of strategies used to implement smoking reduction ^19^. Observational studies have reported higher abstinence rates in smokers who quit abruptly compared to those who quit gradually ^27,28^, though it is possible that other factors contributed to the differences in these studies such as differences in the amount of behavioral support received, type of reduction used, compliance with reduction, differences in levels of dependence, self-efficacy, or motivation to quit, or self-selection into gradual reduction following past unsuccessful abrupt cessation attempts ^29,30^. Interestingly, one randomized study not only reported lower quit rates in smokers randomized to gradual cessation compared to abrupt, but also reported lower quit rates in smokers who preferred gradual cessation, regardless of the arm to which they were assigned ^31^. A recent meta-analysis of 51 studies did not find evidence of a significant overall difference in abstinence rates between abrupt and gradual cessation, suggesting that gradual reduction remains a viable cessation treatment strategy ^19^.

Scheduled smoking is a gradual reduction method of preparing to quit in which smokers reduce their smoking level by systematically increasing the time between cigarettes using a pre-determined schedule ^32-35^. This is accomplished by spreading the increasingly diminishing number of cigarettes evenly over the waking hours so that as fewer cigarettes are smoked as the time between each cigarette gradually increases. This strategy may help to begin the process of de-coupling smoking behavior with specific smoking triggers because the smoking schedule dictates when the cigarette is smoked and prohibits smoking off-schedule. As a result, smokers may not be able to smoke when they want to and might be asked to smoke at times that aren’t ideal or convenient. Scheduled reduction may compel smokers to use coping strategies to overcome the urge to smoke even before the quit day ^22^, skills that may ultimately contribute to long-term abstinence.

In an early randomized treatment study from our lab that compared scheduled smoking to abrupt cessation and a more traditional non-scheduled gradual reduction approach (participants attempted to reduce prior to the quit day with no set schedule), the highest abstinence rates were found in the scheduled smoking group, followed by the abrupt cessation group and then the non-scheduled gradual reduction group. In addition, scheduled reduced smoking was associated with reduced tension, fatigue, urge to smoke, and withdrawal symptoms as well as increased self-efficacy, potentially suggestive of a reduced vulnerability to relapse ^33^. All participants in this study received behavioral counseling and compliance with the smoking schedule was over 75%. Scheduled smoking was also associated with higher abstinence rates and greater cigarette reduction (for those who were unable to quit) in a pilot study with pregnant smokers ^36^. However, in a study with newly diagnosed cancer patients advised to quit by their surgical oncologists, abstinence rates in the scheduled smoking group were not superior to the group with no pre-cessation reduction. It should be noted, however, that both groups in this study received behavioral counseling and pharmacotherapy, and compliance with the smoking schedule was relatively low (<50%) ^32^. Overall, preliminary evidence suggests that scheduled smoking may be an effective way to implement gradual reduction, but schedule compliance may be key in its success, since poor compliance would fail to differentiate this technique from any other method of gradual reduction. For smokers who are unable or unwilling to quit abruptly, providing scheduled smoking as an option could increase the likelihood that they will attempt to quit and take advantage of evidence-based cessation support such as medication or behavioral counseling ^37^.

### Use of Pre-cessation NRT

Nicotine replacement therapy (NRT) was formulated to partially replace the nicotine that would have been consumed in a cigarette without the additional harmful components of combustible tobacco ^38^, thereby reducing the severity of withdrawal symptoms and increasing the likelihood of sustained abstinence ^39^. The effectiveness of NRT has been well-established ^40,41^ and its use generally doubles the likelihood of successful quitting. Despite this, overall abstinence rates among smokers who use NRT, while consistent, are relatively modest (20-30%) ^42,43^. NRT was originally designed to be used after quitting smoking (starting on the quit day), but studies have investigated it’s use during the pre-cessation period, when a smoker often attempts to gradually reduce prior to the quit day.

Exposure to NRT prior to the quit date may allow smokers to acclimate to the effects of the medication and adjust dosage if needed, cut down smoking rate and gain confidence as the quit date approaches, or reduce the reinforcing properties of smoking by initiating nicotine intake separate from smoking ^43^. One study reported that abstinence rates were almost doubled by initiating NRT (patch) two weeks before the target quit date ^44^, but other researchers have reported conflicting data ^43^. A recent meta-analysis summarizing evidence from nine studies comparing NRT use with no NRT use before the quit day (while smoking concurrently) found a positive statistically significant effect of NRT preloading on abstinence (RR=1.25, 95% CI: 1.08 to 1.44; 9 studies, 4395 participants) and concluded that there is moderate-certainty evidence that using NRT prior to quitting improves quit rates. However, the authors noted that more research is needed as many of the included studies may be at high risk for bias ^45^. Studies in smokers with no intention or are unable to attempt quitting abruptly have also indicated that pre-cessation NRT is an effective intervention in achieving sustained smoking abstinence ^46-48^. Importantly, most of the evidence from this meta-analysis comes from studies that included regular behavioral support and monitoring, so it is unclear whether using NRT without regular contact would be as effective ^21^.

### Compliance

The public health impact of smoking cessation treatments (i.e., the number of smokers who successfully quit) depends on the uptake of treatments in the smoking population as well as their effectiveness in actual real-world use ^49^. Previous smoking cessation research has demonstrated that adherence to treatment components both in the delivery from the provider’s perspective (fidelity), and the adherence to dosing, schedules, goals, etc., from the client’s perspective, can impact success in increasing abstinence ^22,32,50-52^. Evidence from a meta-analysis of clinical studies suggests that the lack of adherence to NRT regimens undermines their effectiveness ^53^. Relatively higher smoking cessation rates have been reported in clinical trial participants (50-60%) ^41^ compared to participants of population-based studies (20 to 30%) ^42,43^. Not surprisingly, the rate of adherence to NRT was found to be more than two fold higher in participants of clinical trials compared to participants of population-based studies ^52^. Similarly, as noted above, differences in scheduled smoking adherence may have contributed to differences in the effect of scheduled smoking on abstinence in previous studies ^32,33^. Hence the examination of intervention effectiveness, particularly when two or more interventions are combined (e.g., pre-cessation NRT and Scheduled smoking), should take compliance into account.

### Aims of the current study

The aims of the current study were 1) to evaluate the overall effectiveness of scheduled smoking alone, or in combination with pre-cessation NRT, vs. standard NRT starting on the quit-date with no prior smoking reduction; 2) assess and evaluate the impact of schedule compliance on the effectiveness of the intervention.

## **Methods**

**Figure S1. Sample Schedule Statistics.**


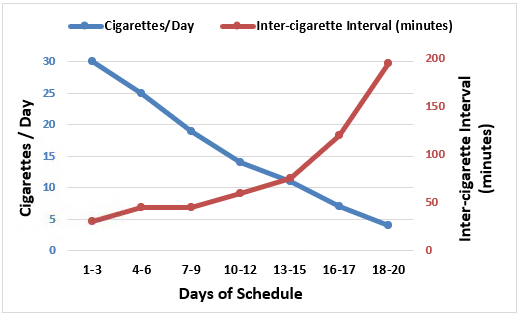


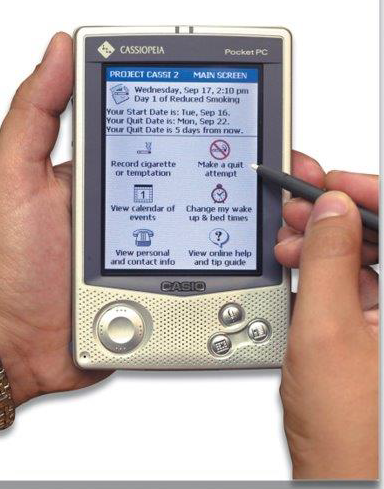


**Figure S2. Handheld Device Delivering the Intervention and Assessments.**


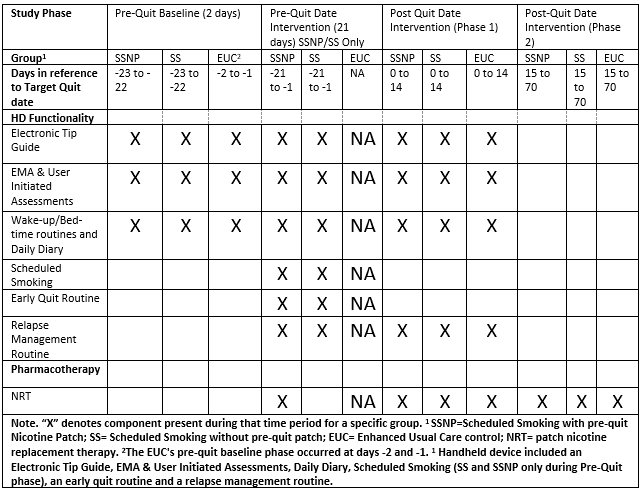


**Figure S3. HD Functionality and Pharmacotherapy over time for each Treatment Group.**

**Figure S4. Tip Guide Topics.**


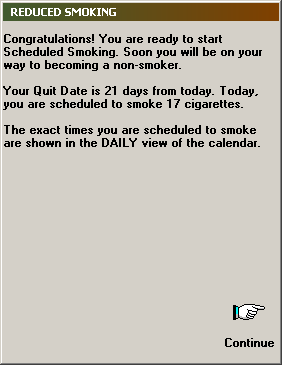

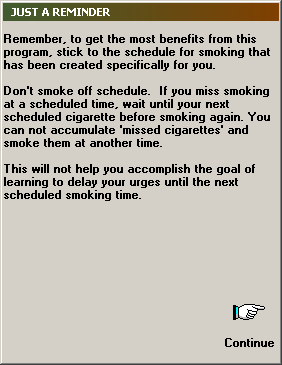

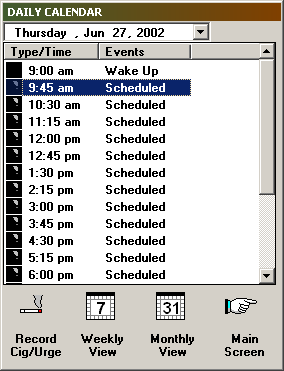


**Figure S5. Screen Shot of First Scheduled Smoking Day.**


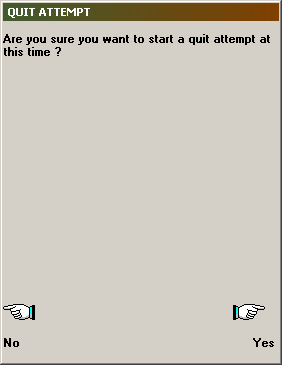

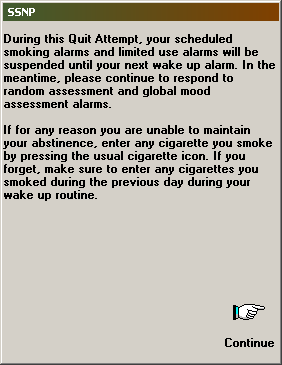

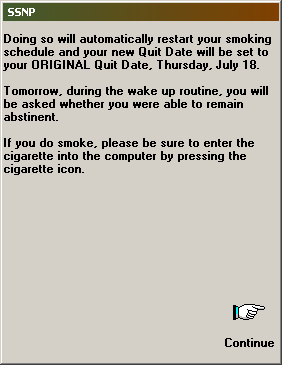


**Figure S6. Sample Screen Shot for an early quit attempt in the scheduled smoking groups.**


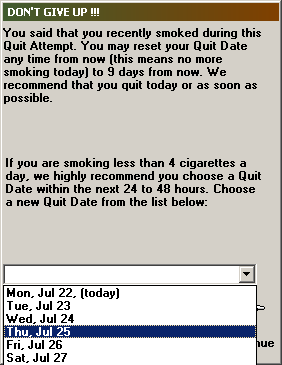

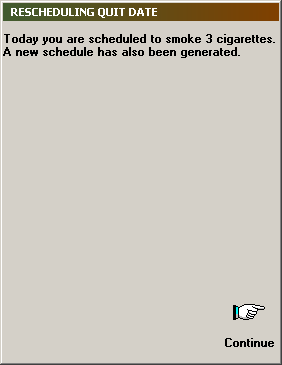


**Figure S7. Sample Screen Shot for resetting the quit date after the original quit date. Panel 1 applies to all groups; Panel 2 on to the scheduled smoking groups.**

# Results

**Table S1** presents the results for both the direct time-dependent effects of treatment on the outcomes and treatment-by-time interaction effects. For the repeated measures main effects results, we found that the SSNP group experienced significantly lower WSWS withdrawal than EUC throughout the study on anxiety (*b* = - 62, *95% CI*: -1.12 - -0.12), sadness (*b* = - 55, *95% CI*: -1.02 - -0.08), and concentration (*b* = - 37, *95% CI*: -72 - -0.01) subscales. Differences in the rate of change from baseline were also noted among the treatment groups. Specifically, the SS group had a significantly lower rate of change in sleep withdrawal from the EUC group (*b* = -0.44, *95% CI*: -0.78 - -0.09). The SSNP group also reported significantly lower rate of change from EUC in CESD Total Score (*b* = - 0.68, *95% CI*: -1.27 - -0.08). Finally, SSNP reported higher rate of change (decline) in craving compared to the EUC group (*b* = 0.32, *95% CI*: -0.4 - -0.06). No other changes in the PANAS positive or negative subscales, or in self-efficacy, were observed.

**Table S1. Longitudinal Models for Withdrawal and Affect Outcomes.**

|  | **Repeated Measures Main Effects (Ref. = EUC)** | | | |  | **Rate of Change Difference (Ref. = EUC)** | | |
| --- | --- | --- | --- | --- | --- | --- | --- | --- |
| **Outcome** | Treatment | Coef. | 95% CI | P-value |  | Coef. | 95% CI | P-value |
| **Hunger** | SS | 0.060 | -0.50 - 0.62 | .83 |  | -0.26 | -0.53 - 0.0036 | .05 |
|  | SSNP | 0.017 | -0.54 - 0.57 | .95 |  | -0.12 | -0.38 - 0.15 | .39 |
| **Anxiety** | SS | 0.026 | -0.48 - 0.53 | .92 |  | -0.078 | -0.32 - 0.16 | .53 |
|  | SSNP | -0.62 | -1.12 - -0.12 | .02 |  | -0.032 | -0.27 - 0.20 | .79 |
| **Sadness** | SS | -0.29 | -0.76 - 0.18 | .22 |  | 0.078 | -0.14 - 0.30 | .49 |
|  | SSNP | -0.55 | -1.02 - -0.085 | .02 |  | 0.069 | -0.15 - 0.29 | .54 |
| **Anger** | SS | 0.18 | -0.27 - 0.63 | .43 |  | -0.097 | -0.31 - 0.12 | .38 |
|  | SSNP | -0.34 | -0.78 - 0.11 | .14 |  | -0.025 | -0.24 - 0.18 | .81 |
| **Concentration** | SS | 0.017 | -0.34 - 0.37 | .92 |  | -0.099 | -0.27 - 0.074 | .26 |
|  | SSNP | -0.37 | -0.72 - -0.012 | .04 |  | -0.028 | -0.20 - 0.14 | .74 |
| **Craving** | SS | 0.35 | -0.27 - 0.96 | .27 |  | 0.28 | -0.0064 - 0.57 | .06 |
|  | SSNP | -0.49 | -1.10 - 0.12 | .27 |  | 0.32 | 0.041 - 0.60 | .03 |
| **Sleep** | SS | 0.43 | -0.29 - 1.14 | .24 |  | -0.44 | -0.78 - -0.090 | .01 |
|  | SSNP | -0.28 | -0.99 - 0.43 | .44 |  | -0.28 | -0.62 - 0.058 | .11 |
| **CESD Total** | SS | 0.026 | -1.23 - 1.28 | .97 |  | -0.58 | -1.19 - 0.026 | .06 |
|  | SSNP | -0.22 | -1.48 - 1.04 | .73 |  | -0.68 | -1.27 - -0.084 | .03 |
| **PANAS Positive** | SS | 0.28 | -0.77 - 1.34 | .60 |  | 0.086 | -0.43 - 0.60 | .75 |
|  | SSNP | 0.84 | -0.22 - 1.91 | .12 |  | -0.13 | -0.64 - 0.37 | .61 |
| **PANAS Negative** | SS | 0.055 | -0.98 - 1.09 | .92 |  | -0.10 | -0.61 - 0.40 | .68 |
|  | SSNP | 0.15 | -0.90 - 1.19 | .78 |  | -0.033 | -0.53 - 0.46 | .90 |
| **Self-Efficacy** | SS | 0.24 | -0.12 - 0.59 | .19 |  | -0.079 | -0.25 - 0.091 | .36 |
|  | SSNP | 0.12 | -0.24 - 0.47 | .52 |  | -0.088 | -0.25 - 0.078 | .30 |

References

1. World Health Organization. WHO report on the global tobacco epidemic, 2011: warning about the dangers of tobacco - Executive Summary. http://www.who.int/tobacco/global_report/2011/en/. Updated September 26, 2013.

2. American Cancer Society. Cancer Facts and Figures. https://www.cancer.org/research/cancer-facts-statistics/all-cancer-facts-figures/cancer-facts-figures-2017.html. Accessed April 14, 2022.

3. American Cancer Society. Tobacco related cancer fact sheet. http://www.cancer.org/cancer/cancercauses/tobaccocancer/tobacco-related-cancer-fact-sheet. Accessed April 14, 2022.

4. US Department of Health and Human Services. *The Health Consequences of Smoking--50 Years of Progress:* *A Report of the Surgeon General*. Atlanta, GA; 2014. Surgeon General's Report.

5. Babb S, Malarcher A, Schauer G, Asman K, Jamal A. Quitting Smoking Among Adults - United States, 2000-2015. *MMWR Morb.Mortal.Wkly.Rep.* 2017;65(52):1457-1464. doi:10.15585/mmwr.mm6552a1.

6. Creamer MR, Wang TW, Babb S, et al. Tobacco Product Use and Cessation Indicators Among Adults — United States, 2018. *MMWR Morb.Mortal.Wkly.Rep.* 2019;68(45):1013-1019. doi:10.15585/mmwr.mm6845a2.

7. Hughes JR, Keely J, Naud S. Shape of the relapse curve and long-term abstinence among untreated smokers. *Addiction*. 2004;99(1):29-38. doi:10.1111/j.1360-0443.2004.00540.x.

8. Chaiton M, Diemert L, Cohen JE, et al. Estimating the number of quit attempts it takes to quit smoking successfully in a longitudinal cohort of smokers. *BMJ Open*. 2016;6(6):e011045. doi:10.1136/bmjopen-2016-011045.

9. *Smoking Cessation: A Report of the Surgeon General*. Washington (DC); 2020.

10. Fiore MC, Jaen CR, Baker TB, et al. Treating Tobacco Use and Dependence: 2008 Update, Clinical Practice Guideline. PM:18807274. Updated March 4, 2015.

11. Cahill K, Stevens S, Perera R, Lancaster T. Pharmacological interventions for smoking cessation: An overview and network meta-analysis. *Cochrane Database Syst.Rev.* 2013;5:CD009329-CD009329. doi:10.1002/14651858.CD009329.pub2.

12. Hartmann-Boyce J, Livingstone-Banks J, Ordóñez-Mena JM, et al. Behavioural interventions for smoking cessation: An overview and network meta-analysis. *Cochrane Database Syst.Rev.* 2021;1:CD013229. doi:10.1002/14651858.CD013229.pub2.

13. West R, McNeill A, Raw M. Smoking cessation guidelines for health professionals: An update. Health Education Authority. *Thorax*. 2000;55(12):987-999. doi:10.1136/thorax.55.12.987.

14. Farley A, Tearne S, Taskila T, et al. A mixed methods feasibility study of nicotine-assisted smoking reduction programmes delivered by community pharmacists - The RedPharm study. *BMC Public Health*. 2017;17(1):210. doi:10.1186/s12889-017-4116-z.

15. Etter JF, Laszlo E, Zellweger JP, Perrot C, Perneger TV. Nicotine replacement to reduce cigarette consumption in smokers who are unwilling to quit: a randomized trial. *J Clin Psychopharmacol*. 2002;22(5):487-495.

16. Ebbert JO, Hughes JR, West RJ, et al. Effect of varenicline on smoking cessation through smoking reduction: A randomized clinical trial. *JAMA*. 2015;313(7):687-694. doi:10.1001/jama.2015.280.

17. Carpenter MJ, Hughes JR, Keely JP. Effect of smoking reduction on later cessation: A pilot experimental study. *Nicotine Tob.Res.* 2003;5(2):155-162. doi:10.1080/146222003100007385.

18. Caldwell BO, Crane J. Combination Nicotine Metered Dose Inhaler and Nicotine Patch for Smoking Cessation: A Randomized Controlled Trial. *Nicotine Tob.Res.* 2016;18(10):1944-1951. doi:10.1093/ntr/ntw093.

19. Lindson N, Klemperer E, Hong B, Ordóñez-Mena JM, Aveyard P. Smoking reduction interventions for smoking cessation. *Cochrane Database Syst.Rev.* 2019;9:CD013183. doi:10.1002/14651858.CD013183.pub2.

20. Hajek P. Withdrawal-oriented therapy for smokers. *Br.J.Addiction*. 1989;84(6):591-598. doi:10.1111/j.1360-0443.1989.tb03474.x.

21. Moore D, Aveyard P, Connock M, Wang D, Fry-Smith A, Barton P. Effectiveness and safety of nicotine replacement therapy assisted reduction to stop smoking: systematic review and meta-analysis. *BMJ*. 2009;338:b1024.

22. Cinciripini PM, Wetter DW, McClure JB. Scheduled reduced smoking: Effects on smoking abstinence and potential mechanisms of action. *Addict.Behav.* 1997;22(6):759-767.

23. Shiffman S, Hughes JR, Ferguson SG, Pillitteri JL, Gitchell JG, Burton SL. Smokers' interest in using nicotine replacement to aid smoking reduction. *Nicotine Tob.Res.* 2007;9(11):1177-1182. doi:10.1080/14622200701648441.

24. Hughes JR, Callas PW, Peters EN. Interest in gradual cessation. *Nicotine Tob.Res.* 2007;9(6):671-675.

25. Abroms LC, Westmaas JL, Bontemps-Jones J, Ramani R, Mellerson J. A content analysis of popular smartphone apps for smoking cessation. *Am J Prev Med*. 2013;45(6):732-736. doi:10.1016/j.amepre.2013.07.008.

26. Hughes JR, Solomon LJ, Fingar JR, Naud S, Helzer JE, Callas PW. The natural history of efforts to stop smoking: A prospective cohort study. *Drug Alcohol Depend*. 2013;128(1-2):171-174. doi:10.1016/j.drugalcdep.2012.08.010.

27. Cheong Y, Yong H-H, Borland R. Does how you quit affect success? A comparison between abrupt and gradual methods using data from the International Tobacco Control Policy Evaluation Study. *Nicotine Tob.Res.* 2007;9(8):801-810. doi:10.1080/14622200701484961.

28. West R, Brown J. Smoking and Smoking Cessation in England 2011: Findings from the Smoking Toolkit Study. http://www.smokinginengland.info/. Updated April 15, 2012. Accessed June 1, 2021.

29. Tan J, Zhao L, Chen H. A meta-analysis of the effectiveness of gradual versus abrupt smoking cessation. *Tob.Induc.Dis.* 2019;17:9. doi:10.18332/tid/100557.

30. Peters EN, Hughes JR, Callas PW, Solomon LJ. Goals indicate motivation to quit smoking. *Addiction*. 2007;102(7):1158-1163. doi:10.1111/j.1360-0443.2007.01870.x.

31. Lindson-Hawley N, Banting M, West R, Michie S, Shinkins B, Aveyard P. Gradual Versus Abrupt Smoking Cessation: A Randomized, Controlled Noninferiority Trial. *Ann.Intern.Med.* 2016;164(9):585-592. doi:10.7326/M14-2805.

32. Ostroff JS, Burkhalter JE, Cinciripini PM, et al. Randomized trial of a presurgical scheduled reduced smoking intervention for patients newly diagnosed with cancer. *Health. Psychol.* 2014;33(7):737-747. doi:10.1037/a0033186.

33. Cinciripini PM, Lapitsky L, Seay S, Wallfisch A, Kitchens K, van Vunakis H. The effects of smoking schedules on cessation outcome: Can we improve on common methods of gradual and abrupt nicotine withdrawal? *J Consult.Clin.Psychol.* 1995;63(3):388-399. doi:10.1037/0022-006X.63.3.388.

34. Cinciripini PM, Lapitsky LG, Wallfisch A, Mace R, Nezami E, van Vunakis H. An evaluation of a multicomponent treatment program involving scheduled smoking and relapse prevention procedures: Initial findings. *Addict.Behav.* 1994;19(1):13-22. doi:10.1016/0306-4603(94)90047-7.

35. Jerome A, Perrone R, Kalfus G. Computer-assisted smoking treatment: A controlled evaluation and long-term follow-up. *J Adv. Med.* 1992;5(1):29-41.

36. Pollak KI, Lyna P, Bilheimer A, et al. A pilot study testing SMS text delivered scheduled gradual reduction to pregnant smokers. *Nicotine Tob.Res.* 2013;15(10):1773-1776.

37. Lindson N, Klemperer EM, Aveyard P. Is there a place for cutting-down-to-stop in smoking cessation support? *Addiction*. 2020;115(10):1797-1799. doi:10.1111/add.14928.

38. McNeill A, Robson D. A man before his time: Russell's insights into nicotine, smoking, treatment and curbing the smoking problem. *Addiction*. 2018;113(4):759-763. doi:10.1111/add.14043.

39. West R, Shiffman S. Effect of oral nicotine dosing forms on cigarette withdrawal symptoms and craving: A systematic review. *Psychopharmacology (Berl)*. 2001;155(2):115-122. doi:10.1007/s002130100712.

40. Silagy C, Lancaster T, Stead L, Mant D, Fowler G. Nicotine replacement therapy for smoking cessation. *Cochrane Database Syst.Rev.* 2004;3:CD000146-CD000146.

41. Hartmann-Boyce J, Chepkin SC, Ye W, Bullen C, Lancaster T. Nicotine replacement therapy versus control for smoking cessation. *Cochrane Database Syst.Rev.* 2018;5:CD000146. doi:10.1002/14651858.CD000146.pub5.

42. Stead LF, Perera R, Bullen C, Mant D, Lancaster T. Nicotine replacement therapy for smoking cessation. *Cochrane Database Syst.Rev.* 2008;1:CD000146-CD000146. doi:10.1002/14651858.CD000146.pub3.

43. Carpenter MJ, Jardin BF, Burris JL, et al. Clinical strategies to enhance the efficacy of nicotine replacement therapy for smoking cessation: a review of the literature. *Drugs*. 2013;73(5):407-426.

44. Rose JE, Herskovic JE, Behm FM, Westman EC. Precessation treatment with nicotine patch significantly increases abstinence rates relative to conventional treatment. *Nicotine Tob.Res.* 2009;11(9):1067-1075.

45. Lindson N, Chepkin SC, Ye W, Fanshawe TR, Bullen C, Hartmann-Boyce J. Different doses, durations and modes of delivery of nicotine replacement therapy for smoking cessation. *Cochrane Database Syst.Rev.* 2019;4:CD013308. doi:10.1002/14651858.CD013308.

46. Batra A, Klingler K, Landfeldt B, Friederich HM, Westin A, Danielsson T. Smoking reduction treatment with 4-mg nicotine gum: A double-blind, randomized, placebo-controlled study. *Clin Pharmacol Ther*. 2005;78(6):689-696. doi:10.1016/j.clpt.2005.08.019.

47. Wennike P, Danielsson T, Landfeldt B, Westin A, Tonnesen P. Smoking reduction promotes smoking cessation: results from a double blind, randomized, placebo-controlled trial of nicotine gum with 2-year follow-up. *Addiction*. 2003;98(10):1395-1402.

48. Rennard SI, Glover ED, Leischow S, et al. Efficacy of the nicotine inhaler in smoking reduction: A double-blind, randomized trial. *Nicotine Tob.Res.* 2006;8(4):555-564. doi:10.1080/14622200600789916.

49. Shiffman S, Gitchell J, Pinney JM, Burton SL, Kemper KE, Lara EA. Public health benefit of over-the-counter nicotine medications. *Tob Control*. 1997;6(4):306-310. doi:10.1136/tc.6.4.306.

50. Lorencatto F, West R, Christopherson C, Michie S. Assessing fidelity of delivery of smoking cessation behavioural support in practice. *Implement. Sci.* 2013;8:40. doi:10.1186/1748-5908-8-40.

51. Lindqvist H, Forsberg L, Enebrink P, Andersson G, Rosendahl I. The relationship between counselors' technical skills, clients' in-session verbal responses, and outcome in smoking cessation treatment. *J Subst Abuse Treat*. 2017;77:141-149. doi:10.1016/j.jsat.2017.02.004.

52. Mersha AG, Eftekhari P, Bovill M, Tollosa DN, Gould GS. Evaluating level of adherence to nicotine replacement therapy and its impact on smoking cessation: A systematic review and meta-analysis. *Arch. Public Health*. 2021;79(1):26. doi:10.1186/s13690-021-00550-2.

53. Raupach T, Brown J, Herbec A, Brose L, West R. A systematic review of studies assessing the association between adherence to smoking cessation medication and treatment success. *Addiction*. 2014;109(1):35-43. doi:10.1111/add.12319.
